# Using nutritional geometry to define the fundamental macronutrient niche of the widespread invasive ant *Monomorium pharaonis*

**DOI:** 10.1371/journal.pone.0218764

**Published:** 2019-06-20

**Authors:** Birla A. Krabbe, Xavier Arnan, Pol Lannes, Christoffer Echtvad Bergstedt, Rasmus Stenbak Larsen, Jes Søe Pedersen, Jonathan Z. Shik

**Affiliations:** 1 Section for Ecology and Evolution, Department of Biology, University of Copenhagen, Copenhagen, Denmark; 2 CREAF, Cerdanyola del Vallès, Spain; Colorado State University, UNITED STATES

## Abstract

The emerging field of nutritional geometry (NG) provides powerful new approaches to test whether and how organisms prioritize specific nutritional blends when consuming chemically complex foods. NG approaches can thus help move beyond food-level estimates of diet breadth to predict invasive success, for instance by revealing narrow nutritional niches if broad diets are actually composed of nutritionally similar foods. We used two NG paradigms to provide different, but complementary insights into nutrient regulation strategies and test a hypothesis of extreme nutritional generalism in colony propagules of the globally distributed invasive ant *Monomorium pharaonis*. First, in two dimensions (protein:carbohydrates; P:C), *M*. *pharaonis* colonies consistently defended a slightly carbohydrate-biased intake target, while using a generalist equal-distance strategy of collectively overharvesting both protein and carbohydrates to reach this target when confined to imbalanced P:C diets. Second, a recently developed right-angled mixture triangle method enabled us to define the fundamental niche breadth in three dimensions (protein:carbohydrates:lipid, P:C:L). We found that colonies navigated the P:C:L landscape, in part, to mediate a tradeoff between worker survival (maximized on high-carbohydrate diets) and brood production (maximized on high-protein diets). Colonies further appeared unable to avoid this tradeoff by consuming extra lipids when the other nutrients were limiting. Colonies also did not rely on nutrient regulation inside their nests, as they did not hoard or scatter fractions of harvested diets to adjust the nutritional blends they consumed. These complementary NG approaches highlight that even the most successful invasive species with broad fundamental macronutrient niches must navigate complex multidimensional nutritional landscapes to acquire limiting macronutrients and overcome developmental constraints as small propagules.

## Introduction

The factors enabling a species to thrive outside its native range include propagule pressure [1], the absence of natural enemies [2], and the ecological match between its introduced and native habitat [3,4]. Invasive establishment is also governed by a species’ ability to acquire nutritionally suitable food following introduction [5,6]. Despite this simple premise, foods are actually complex mixtures of water, fibers, macronutrients (i.e. proteins, carbohydrates, and lipids), micronutrients (e.g. Na, P, K), toxins, and vitamins [7,8]. And, while theory [9,10] and empirical data [11] support that invasive species tend to be dietary generalists, few studies have revealed the specific nutritional dimensions defining this wide dietary niche breadth [12,13]. Nutritional constraints are likely to be especially important during early phases of an invasion since organisms must typically survive a bottleneck of small population size, before other behavioral and life history traits conferring invasive success can be expressed.

In recent years, nutritional geometry (NG) has become a powerful hypothesis-driven approach for visualizing how taxa from slime molds [14] to gorillas [15] prioritize multiple competing nutritional requirements to maximize their fitness [7]. Since these analyses explicitly define foods as mixtures of multiple covarying nutrients, it is possible to test the hypothesis that successful invaders have exceptionally wide physiological tolerances for nutritionally imbalanced foods, while describing an organism’s ‘fundamental macronutrient niche’ in several dimensions of co-varying nutritional availability [12]. In the present study, we explore multidimensional nutritional requirements and strategies for meeting those requirements in the invasive ant *Monomorium pharaonis*. We do this by employing two different, but complementary NG paradigms: *1)* a 2-D protein:carbohydrate (P:C) approach to visualize how and why colonies prioritize specific nutrients when prevented from reaching their self-selected optimum, and *2)* a 3-D protein:carbohydrate:lipid (P:C:L) approach to map the fundamental macronutrient niche of *M*. *pharaonis*.

Thought to be the world’s most widely distributed non-native ant species, *M*. *pharaonis* has been found in so many regions for so many years that its native habitat in Asia has only recently been triangulated [16]. Populations of *M*. *pharaonis* are nearly entirely limited to the human-built world, where they can access plentiful foods and stable abiotic conditions, and where they rarely displace native species [17]. And, while *M*. *pharaonis* also exhibits a suite of life history traits (i.e. polygyny and polydomy) favoring its ecological success as massive colonies [18], these traits are likely expressed only when colonies reach large sizes, after propagules have survived founding bottlenecks. Below, we describe the NG framework used in the present study, focusing on the nutritional dimensions enabling small propagules to rapidly increase their colony size.

**Fig 1:**
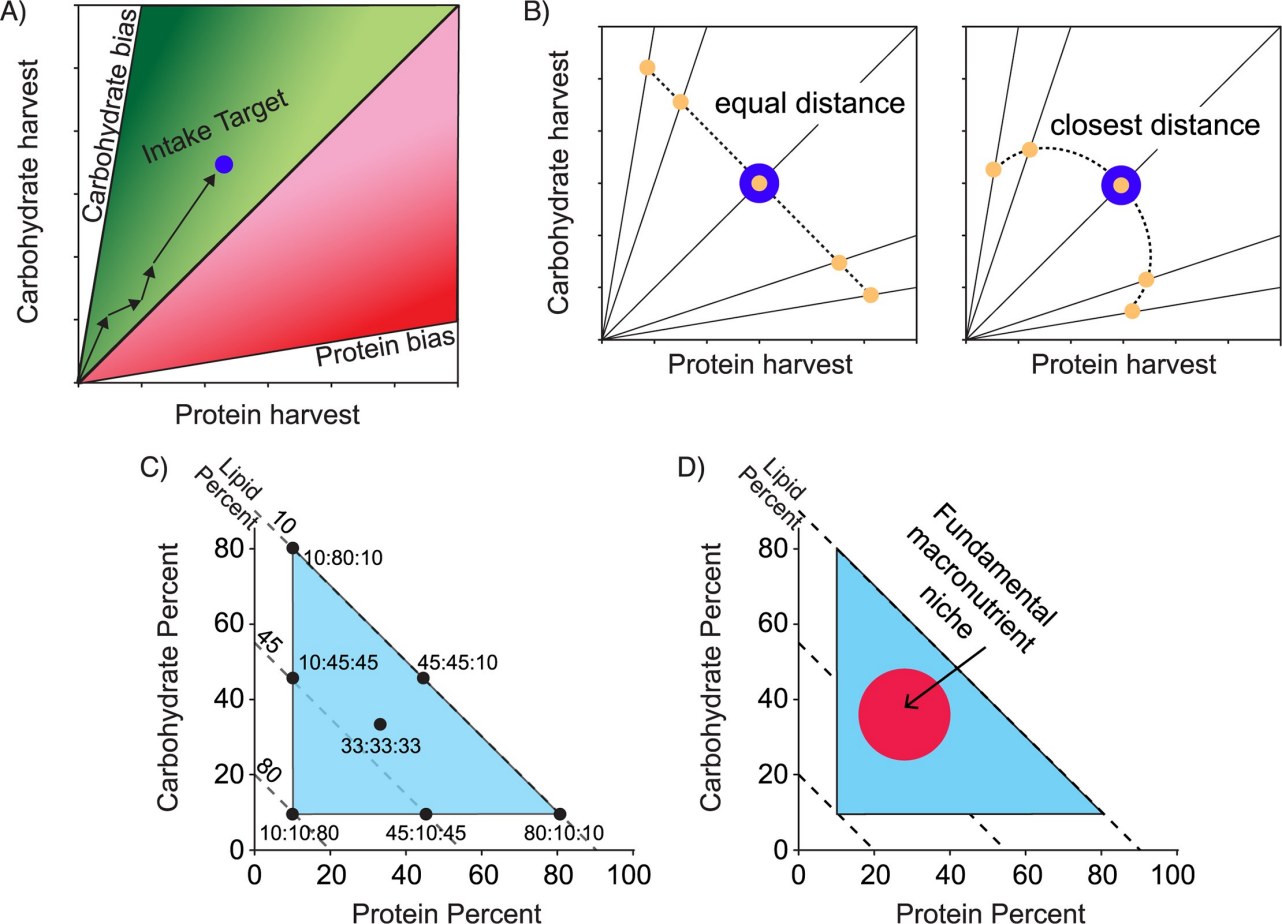
Schematic figure showing how 2-D and 3-D nutritional geometry approaches can identify strategies for prioritizing competing nutritional needs. (A) In 2-D choice experiments, animals reveal their intake target as the nutritional blend that maximizes performance (blue dot). Here, an animal has consumed four successive meals, switching between carbohydrate-biased and protein-biased diets to reach a slightly carbohydrate-biased intake target (adapted from [19]). (B) In 2-D no-choice experiments, animals reveal a rule of compromise intake array when confined to single nutritionally imbalanced diets that constrain their intake P:C ratio to ‘nutritional rails’ extending from the origin. Here, colonies were confined to 5 P:C diets and cumulative intake values were measured for each diet treatment (orange dots). Dashed lines connect these intake values and reflect decisions about over-harvesting one macronutrient to avoid under-harvesting a more crucial limiting macronutrient relative to the intake target (blue dot). Generalist consumers tend to obey the ‘equal distance rule’, with a straight-line array, as they expect to redress a temporary imbalance by switching to another complementary food later. Specialist consumers tend to obey the ‘closest distance rule’, with a convex array reflecting efforts to stay as close to the intake target as possible (adapted from [7]). (C) Using 3-D nutritional landscapes, a fundamental macronutrient niche can be visualized with three nutrient mixtures shown in bivariate plots [22]. Here, each black dot is a specific mixture of protein, carbohydrates, and lipids (P:C:L). Protein and carbohydrate values are plotted on X and Y axes respectively, and lipids are plotted as diagonal lines with negative slopes that intersect P:C:L points with the sum of the 3 nutrients adding to 100%. In this example, organisms are provided seven P:C:L diets (e.g. 10:10:80 has 10% P + 10% C + 80% L) in a no-choice experiment. Diet harvest and performance are then mapped by interpolating between values measured at each diet (adapted from [23]). (D) We can define the fundamental macronutrient niche (FMN) by mapping regions of maximal diet harvest and/or consumption across the P:C:L landscape (adapted from [12]).

NG studies typically focus on foraging tradeoffs made when organisms are confined in laboratory experiments to chemically defined diets with two co-varying nutrients, usually protein and carbohydrates [19]. In such 2-D choice experiments, organisms feed between two diets with imbalanced protein:carbohydrate (P:C) ratios, selecting their own intake target, defined as the blend of protein and carbohydrates that maximizes their performance (Fig 1A). In no-choice experiments, organisms are confined to single diets and move up a nutritional rail reflecting that diet’s specific P:C ratio (Fig 1B). We can then connect intake values across no-choice diet treatments to visualize a rule of compromise (Fig 1B) that reflects a strategy to approach the intake target by over-eating or under-eating one macronutrient to acquire the other limiting nutrient.

Rules of compromise can be used to infer the degree of macronutrient specialization [7]. Generalists (e.g. cockroaches; [20]) tend to exhibit the equal-distance rule, with diagonal intake arrays indicating similar excess harvest of both macronutrients to reach the intake target for the other limiting macronutrient (Fig 1B). Macronutrient specialists (e.g. host-specific herbivores; [21]) typically exhibit the closest-distance rule with curved intake arrays indicating inflexible intake of one or both imbalanced nutrients relative to the intake target (Fig 1B). While equal-distance arrays reflect the generalist forager expectation of finding other complementary foods in the environment to redress temporary nutritional imbalance, closest-distance arrays reflect that specialists lack such an expectation since they only consume a single food type [19].

NG also provides a graphical framework for visualizing intake and performance across three-component dietary mixtures (e.g. protein, carbohydrates, and lipids; [22]). This approach uses nutritional landscapes to visualize an organism’s fundamental macronutrient niche (hereafter FMN) as the area where introduced populations could successfully establish by consuming only that blend of macronutrients [12] (Fig 1C). In laboratory experiments, the 3-D approach involves a no-choice experiment with several chemically-defined protein:carbohydrate:lipid (P:C:L) diets that yield macronutrient maps of intake and performance [23] (Fig 1C). Nutritional landscapes highlight likely invasive species as those with intrinsically broader FMN that improve the odds of acquiring nutritionally suitable foods in an introduced habitat [12] (Fig 1D).

Most ant species are food generalists, opportunistically foraging widely within broad macronutrient categories, for example scavenging across species of protein-rich prey items [24]. However, ant colonies also reliably harvest specific macronutrient intake targets in feeding experiments [25–27]. We thus used a 2-D P:C feeding experiment with *M*. *pharaonis* propagules to first visualize whether it defends an intake target, and then test the prediction of a generalist equal-distance intake array. We next used a 3-D approach to visualize the FMN for the first time for any ant species, exploring whether and how *M*. *pharaonis* colonies selectively forage across a P:C:L landscape. By comparing maps of diet consumption and colony performance, we further tested whether colonies face tradeoffs between brood production (predicted to be fueled by protein) and adult worker maintenance (predicted to depend on carbohydrates) as they increase colony size from small introduced propagules [28,29], and whether lipids can provide an alternative non-protein energy source mediating a growth-survival tradeoff [23]. Since ant colonies can selectively store and dispose of harvested nutrients [25,30], we also used colored diets to test a hypothesis of post-harvest nutrient regulation, determining whether harvested macronutrients were differentially consumed, hoarded (inside the nest), or scattered (outside the nest).

## Methods

### Experimental setup

We established *M*. *pharaonis* propagules generated from a lab population of ants originally collected from eight source populations that span the global distribution of this species in Florida (n = 2 populations), Texas (n = 2 populations), Malaysia (n = 1), London (n = 1), Warsaw (n = 1), and Ghana (n = 1) [31,32]. For over a decade, a blended stock colony made by mixing ants from all eight source populations has been maintained in many plastic bins on sucrose-agar diet and freshly killed crickets in climate-controlled rooms at 27°C and 50% R.H (see S1 Appendix for detailed methods). All experimental colonies described below are composed of ants blended from the eight source populations, but maintained in separate plastic bins (n = 6 bins 2-D experiment, n = 8 bins 3-D experiment). We removed ants from bins with a small paintbrush and combined them into petri dishes where they rapidly relocated to nesting areas. During a brief acclimation period prior to feeding experiments, newly formed colonies were fed *ad lib* amounts of sucrose-agar diet. During feeding experiments, lids containing pre-weighed experimental foods were placed in the foraging area near the nest, along with *ad lib* water. Diets were modified versions of a published protein:carbohydrate (P:C) [33] and a protein:carbohydrate:lipid (P:C:L) diet [23]. Recipes for 2-D P:C diets (S1 Table) were standardized for total macronutrient concentrations (100 g/L), while manipulating macronutrient ratios, since protein and carbohydrates contain roughly similar amounts of energy on a per-gram basis [33]. The 3-D P:C:L diet recipes (S2 Table) were standardized for total energy content across diets (*ca*. 675 joules), while manipulating the relative energy content (joules) provided by each macronutrient (Fig 1C, *as per* [23]), since lipids contain roughly twice the amount of energy as carbohydrates and proteins [34]. These P:C:L diets contained lipids in a 4:1:1:1:1 ratio of lard:fish-oil:sunflower-oil:rapeseed-oil:peanut-oil, selected based on pilot cafeteria-style feeding experiments showing strongest recruitment to lard and similar (but lower) recruitment to the four selected oils, relative other available options (S1 Appendix).

We measured diet harvest by placing *ca*. 1-cm^3^ pre-weighed (initial wet mass) diet cubes inside colony foraging areas each day, and then oven-drying these and cubes of control diet at 60°C for 24 hours. We then weighed the dry diets to the nearest 1 μg (AG285 Mettler Toledo) and calculated cumulative diet harvest across days of the experiment (S1 Appendix). We also added food coloring (Dr. Oetker ^TM^) to each diet just prior to blending all ingredients, so we could separate it from debris when collecting hoarded (piled within the defined nest area) and scattered (discarded in the foraging area) diet at the ends of feeding experiments. Consumed diet was harvested diet minus the summed mass of hoarded and scattered diet.

### 2-D nutritional geometry experiment

We performed a choice experiment with two separate choice combinations (1:6 vs. 3:1 or 1:3 vs. 6:1 P:C) to test whether colonies reliably selected the same intake target (Fig 1A). We also performed a no-choice experiment (confining colonies to single 1:6, 1:3, 1:1, 3:1, or 6:1 P:C diets) to measure foraging along intake rails and determine the shape of the intake array (Fig 1B). We assembled 64 colonies, each with 200 workers and a scoop (0.5 x 0.5 x 0.15 cm) of brood and collected dead workers from each colony during a 4-day acclimation period, replacing them on day 1 of the experiment to standardize initial colony size. We assigned 24 colonies to the choice experiment (n = 12 colonies per choice pairing treatment and removed one colony from each choice treatment due to missing intake data) and 40 colonies to the no-choice experiment (n = 8 colonies per diet treatment). Over 12 days in both choice and no-choice experiments, we replaced old diet with fresh each day, counted and collected dead workers every fourth day during the experiment, and counted the remaining living workers on day 12 (S1 Appendix).

### 3-D nutritional geometry experiment

We established 35 colonies, each with 200 workers and four queens, and let them initiate brood production during an acclimation period of from 9 to 11 days. Just before day 1 of the experiment, we replaced any dead workers and assigned colonies to one of seven P:C:L diets (33:33:33, 80:10:10, 10:80:10, 10:10:80, 45:45:10, 10:45:45, 45:10:45) in a no-choice experiment (n = 5 colonies per diet treatment) (Fig 1C). Over 14 days, we replaced diet and collected and counted dead workers each day. Following the experiment, we counted and weighed (dry mass) pupae, larvae, and workers and counted all eggs. This experiment length enabled us to accurately measure adult worker mortality, since egg to adult durations can vary from 22 to 54 days (i.e. maturing brood were unlikely to replace dead workers [35]), but also precluded meaningful analysis of larvae and pupae production, since too few eggs reached these advanced developmental stages over 14 days (S1 Appendix).

### Statistical analyses

#### 2-D analyses

We used the R v.3.2.4 statistical environment [36] to perform all statistical analyses. We first used ANOVAs testing for differences in the response variables harvested diet, consumed diet, hoarded diet, and scattered diet across the categorical explanatory variable of diet treatment in separate analyses for the choice experiment (1:6 + 3:1 P:C vs. 1:3 + 6:1 P:C) and the no-choice experiment (1:6, 1:3, 1:1, 3:1, 6:1 P:C). The response variables of harvested and consumed diet were further subdivided into total, protein, and carbohydrate amounts, based on calculations from the defined P:C recipes [33]. Significant main effects were followed with post-hoc Tukey tests. In the choice experiment, we further analyzed the total amounts of protein and carbohydrates that were hoarded and scattered by colonies (based on diet recipes). We further used paired t-tests (four separate analyses for two choice pairing treatments and two response variables) to test the null hypothesis that colonies hoarded and/or scattered the same mass of the carbohydrate-biased or protein-biased diet, fragments of which could be sorted by color. We used the glmer function in the lme4 package to perform generalized linear mixed models with a binomial distribution and a logit link function with separate analyses for the choice and no-choice experiments examining how the response variable proportion of living workers (alive / (alive + dead)) varied with the explanatory variables of day, diet treatment, and their interaction. We also performed a combined test comparing the response variable proportion of living workers across choice and no-choice experiments, with the explanatory variables of experiment (choice, no-choice), day, and their interaction. Initial colony bin was added as a random factor.

#### 3-D analyses

We performed GLM analyses testing for differences in foraging response variables (harvested diet, consumed diet, scattered diet, hoarded diet) across the P:C:L diet treatments, further exploring significant results using post-hoc Tukey tests. We then used Response Surface Models (RSM) to analyze the influence of the relative amount of each macronutrient in diets on response variables, identifying relationships between variables via second-degree polynomial models [37,38]. Since the relative amount of each nutrient results from the combination of the other two nutrients (i.e. the summed amounts of the three nutrients always equals one), the three nutrients cannot be included in the same model as explanatory variables. We thus performed separate RSM models for each response variable: the four foraging variables and two performance variables (percent worker survival, egg number), where the linear and quadratic components for protein and carbohydrate intake and the cross-product of protein and carbohydrate were added as explanatory variables. Lipid effects on response variables reflect P:C:L diet recipes, such that high values of either protein or carbohydrates indicate low lipid values, low values of both protein and carbohydrates indicate high lipid values, and low to medium values of both protein and carbohydrates indicate medium lipid values.

We performed these analyses using the function rsm from the rsm package [39], first running complete models. If quadratic terms were not significant, we ran models again without non-significant quadratic terms. To help interpret the patterns, we estimated P:C:L ratios at which the response is maximized by using the function optimx from package optimx [40] in R. For response variables with significant overall RSM models, we then visualized P:C:L landscapes using the fields package [41] to calculate non-parametric thin-plate splines [14,42] and map foraging and performance variables. We set the topological resolution of response surfaces to λ = 0.001 as a smoothing parameter.

## Results

**Fig 2:**
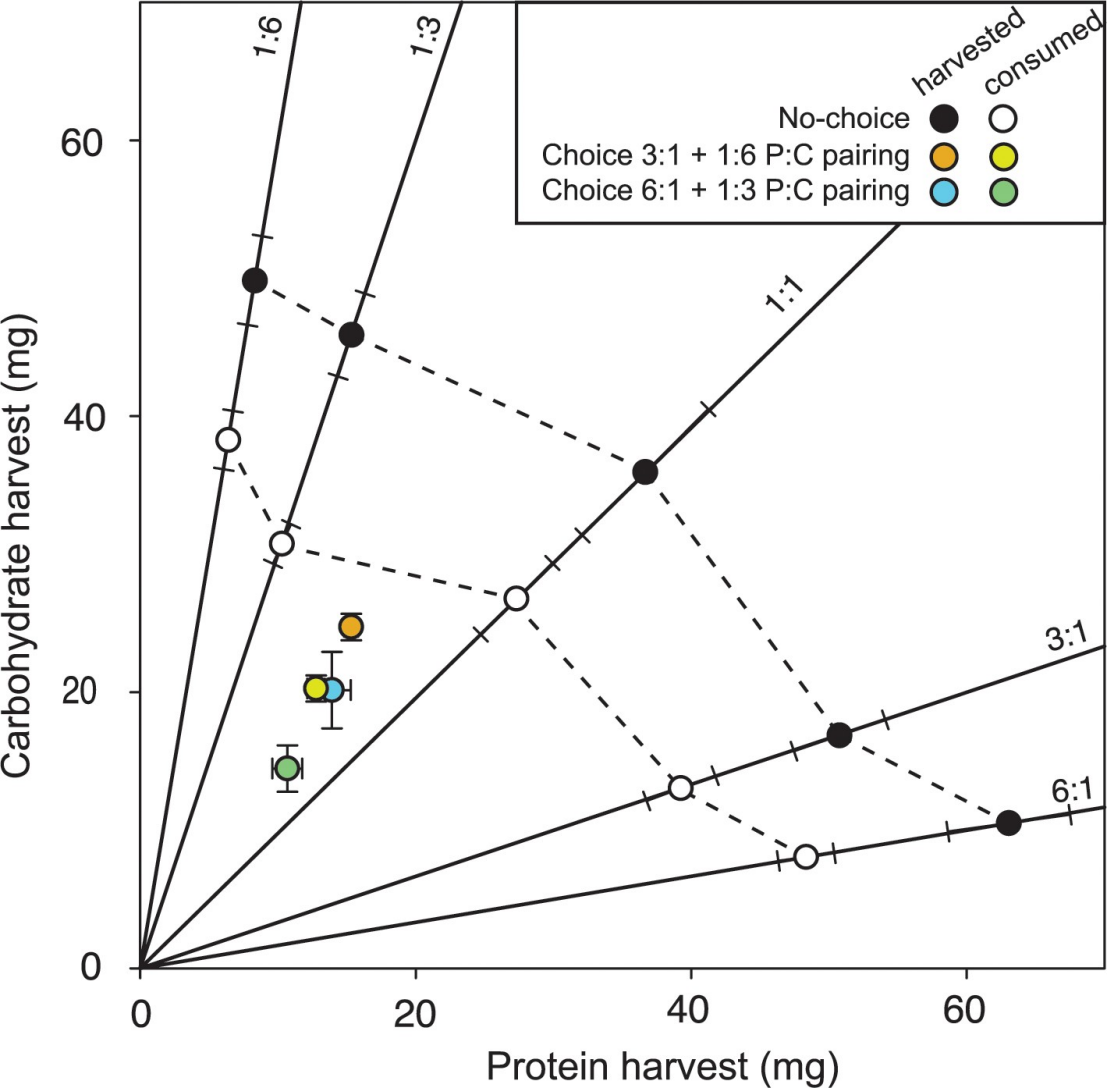
Nutritional geometry in two dimensions. In a choice experiment lasting 12 days, colonies reliably harvested and consumed a slightly carbohydrate-biased 1:1.5 P:C intake target in both choice diet combinations. In a no-choice experiment, colonies exhibited a generalist equal distance intake array (dashed lines) by harvesting similar excesses of protein and carbohydrates relative to their intake target. Diet consumption (subtracting scattered and hoarded amounts from harvested diet) tightly matched diet harvest. Solid black lines show nutrient rails for each no-choice P:C diet treatment. Choice experiment harvest values are provided with bi-directional error bars, and no-choice harvest values provided with pythagorean standard error bars aligned with the intake rail (as per [62]).

**Fig 3:**
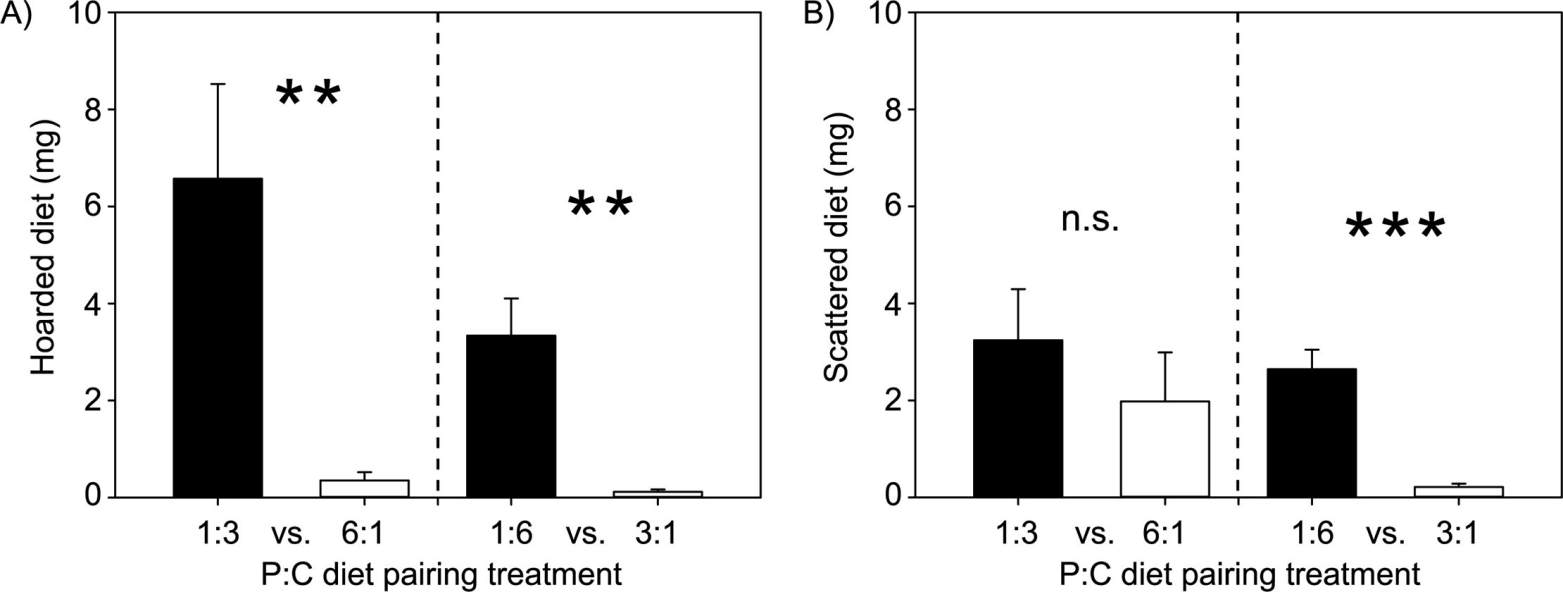
Post-harvest diet processing (± SE) after 12 days in the 2-D choice experiment, based on analyzing uneaten colored diet. (A) Colonies tended to preferentially hoard (retained inside the nest) carbohydrate-biased diets in both the 1:3 vs. 6:1 P:C pairing and the 1:6 vs. 3:1 P:C pairing treatments. (B) Colonies scattered diets evenly in the 1:3 vs. 6:1 P:C treatment, but scattered significantly more of the carbohydrate-biased diet in the 1:6 vs. 3:1 P:C treatment. Asterisks indicate the results of paired t-tests, testing difference within pairings between either hoarded or scattered diet (dry mass, mg), with n.s. = not significant, * p < 0.05, ** p < 0.01, *** p < 0.001).

In the 2-D choice experiment, colonies consistently selected slightly carbohydrate-biased 1:1.5 P:C intake target, harvesting similar amounts of total diet (F_1,20_ = 1.9, p = 0.185), protein (F_1,20_ = 0.9, p = 0.352) and carbohydrates (F_1,20_ = 2.43, p = 0.135) in both choice pairings (Fig 2). Despite similar harvesting strategies, colonies consumed more of their harvested diet when provided the more extreme carbohydrate-biased 1:6 vs. 3:1 P:C choice pairing (F_1,20_ = 12.1, p = 0.002; Fig 2). However, it was not immediately clear whether colonies regulated this consumption by processing nutrients in their nests, since colonies hoarded (F_1,20_ = 2.5, p = 0.131) and scattered (F_1,20_ = 2.7, p = 0.115) similar amounts of their harvested diet in both diet pairings. Within colonies, we next used sorted colored diets to show that colonies preferentially hoarded slightly more carbohydrate-biased diet than protein-biased diet in both 1:3 vs. 6:1 P:C (t_10_ = 3.3, p < 0.007) and 1:6 vs. 3:1 P:C (t_10_ = 4.3, p < 0.001) pairing treatments (Fig 3A), while also hoarding (averaged (± SD) across diet pairing treatments) only 10 ± 7.8% of the diet they harvested. Scattered diet amounts were more variable, being even between diets in the more protein-biased 1:3 vs. 6:1 pairing (t_10_ = 0.8; p = 0.435) while representing only 5.2 ± 2.5% of the harvested diet (Fig 3B), but with carbohydrate-biased diet being more heavily scattered when colonies were provided the more extreme carbohydrate-biased 1:6 vs. 3:1 P:C pairing (t_10_ = 6.3; p < 0.001) with 11.0 ± 8.8% of harvested diet being scattered (Fig 3B).

In the 2-D no-choice experiment, colonies obeyed the equal distance rule (Fig 2), harvesting similar amounts of diet across no-choice treatments (F_4,35_ = 2.0, p = 0.120) (S1 Fig). Colonies thus harvested significantly more carbohydrates when confined to high-carbohydrate diets (1:6, 1:3 P:C) (F_4,35_ = 36.7, p < 0.001; S1 Fig) and significantly more protein on high-protein diets (6:1, 3:1 P:C) (F_4,35_ = 50.3, p < 0.001; S1 Fig). In this way, colonies let both nutrients fluctuate while regulating total diet harvest (Fig 2). Colonies further hoarded (F_4,35_ = 1.0, p = 0.416) and scattered (F_4,35_ = 1.2, p = 0.314) similar amounts of total diet across P:C diet treatments, while hoarding (14.4 ± 9.8%) or scattering (7.8 ± 5.4%) small fractions of their total harvested diet. Although we did not detect nutrient-specific hoarding or scattering behavior, colonies still consumed more of the highest protein 6:1 P:C diet than either of the low-protein diets (1:3 and 1:6 P:C) (F_4,35_ = 5.1, p = 0.002; Fig 2 and S2 Fig). Thus, while colonies confined to no-choice diet treatments over-consumed both carbohydrates (F_4,35_ = 56.0, p = 0.0001; S2 Fig) and protein (F_4,35_ = 93.0, p < 0.001; S2 Fig) relative to the intake target (Fig 2), they over-consumed high-protein diets to a greater degree.

This elevated protein consumption on no-choice diets (especially on 3:1 and 6:1 P:C diets; S2 Fig) was associated with reduced adult worker survival (χ^2^_1_ = 70.4, p = 0.0001), which became more pronounced at later sampling days (χ^2^_1_ = 146.9, p < 0.001) (S3 Fig). Similarly, in the choice experiment, survival was lower in the more protein-biased 1:3 vs. 6:1 P:C pairing treatment relative to the 1:6 vs. 3:1 pairing treatment (χ^2^_1_ = 18.5, p < 0.001; S3 Fig). As expected, when comparing between the no-choice and choice experiments, adult workers had lower mortality over time when they could select their own intake target in the choice experiment relative to when they were confined to a single diet in the no-choice experiment (χ^2^_1_ = 40.9, p < 0.001).

**Fig 4:**
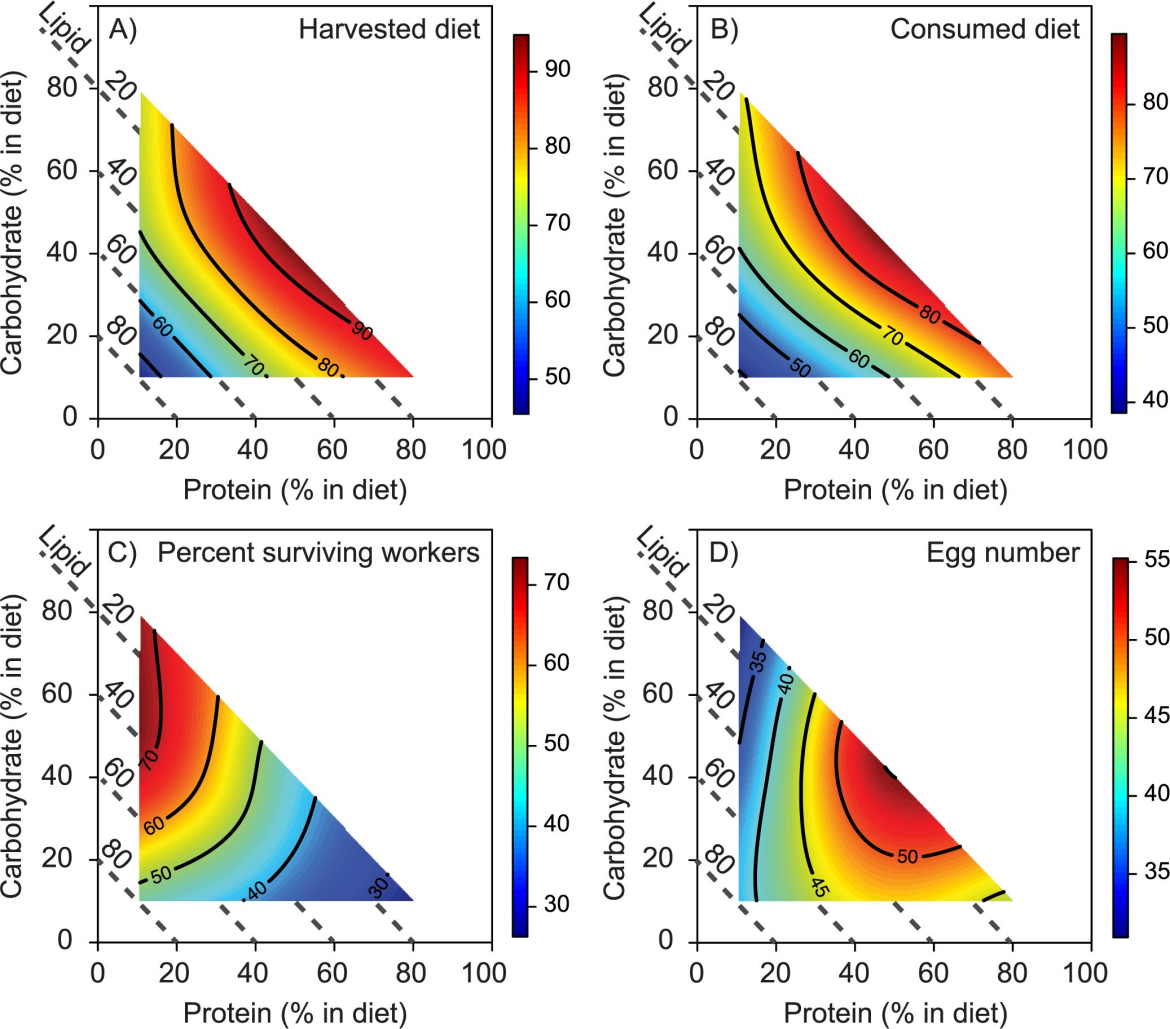
Nutritional landscapes visualize the fundamental macronutrient niche (FMN) of *M*. *pharaonis* in three dimensions (proteins, carbohydrates, and lipids; P:C:L). (A) Colonies harvested maximal amounts across a broad range of protein-biased and carbohydrate-biased diets, while avoiding lipid-biased diets. (B) Colonies consumed most of the diet they harvested regardless of the P:C:L content. (C) The survival percentage of adult workers was highest on diets with moderate to high carbohydrates, low protein and low to moderate lipids. In contrast, moderate to high amounts of protein yielded the lowest worker survival, even as carbohydrates and lipids increased. (D) Colonies produced the most eggs when colonies were confined to diets with similar levels of protein and carbohydrates and low lipid content, and the fewest eggs on all diets where protein availability was low, regardless of carbohydrate and lipid amounts. Isoclines (red areas are highest values and blue areas are lowest) indicate dry mass of diet (mg), percent worker survival, or egg numbers, with scale bars adjusted relative to the range of observed values. Landscapes comprise 7 P:C:L ratios (*see* Fig 1C) and diet percentages (axis labels) indicate energy content provided by each macronutrient, with total energy content available standardized across diets (see Methods). Response surface regressions underlying colored heat maps were significant in each panel (Table 1).

In the 3-D experiment (as in the 2-D experiment), both diet harvest (Fig 4A) and diet consumption (Fig 4B) increased linearly as the relative protein and carbohydrate content increased, with colonies maintaining high consumption levels on the most protein-rich diets, but not on the most carbohydrate-rich diets (Table 1 and S3 Table). Diet nutrients thus had strong effects on diet harvest (explaining 63% of total variation) and diet consumption (explaining 81% of total variation) (Table 1). The fundamental macronutrient niche (FMN) of *M*. *pharaonis*, visualized in the bright red areas across the diet consumption landscape (Fig 4B) spans a wide protein and carbohydrate gradient, with maximal diet consumption (44:56:0 P:C:L, Table 1) occurring at intermediate levels of protein and carbohydrates, but the lowest lipid levels (Fig 4B). As in the 2-D experiment, colonies did not appear to rely on post-harvest nutrient processing to target a narrower region of their harvested diet landscape, since P:C:L diet treatment explained 0% of variation in hoarded diet and 12% of variation in scattered diet, with non-significant RSM models (Table 1). Moreover, most of the harvested diet was consumed (max *ca*. 80 mg) rather than hoarded (max *ca*. 8 mg) or scattered (max *ca*. 4 mg) (S4 Fig and S3 Table).

**Table 1:** Statistical output from Response Surface Models (RSM) analyses of 3-D feeding experiment.

Effect	Response variable	Estimate	Std. Err.	t	p	R^2^	Lack of fit (p)	Maximum response
Harvested diet					<0.0001	0.63	0.307	
	Protein	0.669	0.158	4.2	0.0002			54
	Carbohydrate	1.705	0.536	3.2	0.0034			46
	Protein^2^	-	-	-	ns			
	Carbohydrate^2^	-0.015	0.005	-2.9	0.07			
	Protein*Carbohydrate	-0.000	0.007	-0.02	0.9865			
Consumed diet					<0.0001	0.81	0.897	
	Protein	0.501	0.104	4.8	<0.0001			44.3
	Carbohydrate	1.323	0.354	3.7	0.0008			55.6
	Protein^2^	-	-	-	ns			
	Carbohydrate^2^	-0.011	0.004	-3.3	0.0025			
	Protein*Carbohydrate	0.009	0.005	1.8	0.0835			
Hoarded diet					0.4111	0.00	0.342	
Scattered diet					0.0921	0.12	0.425	
Percent surviving workers					<0.0001	0.55	0.812	
	Protein	0.225	0.557	0.4	0.689			0.0
	Carbohydrate	7.120	1.890	3.8	0.0007			61.8
	Protein^2^	-	-	-	ns			
	Carbohydrate^2^	-0.058	0.019	-3.10	0.0041			
	Protein*Carbohydrate	-0.079	0.025	-3.10	0.0040			
Number of eggs					0.0108	0.23	0.856	
	Protein	-0.166	0.166	-1.0	0.3241			56.8
	Carbohydrate	-0.400	0.170	-2.4	0.0251			43.2
	Protein^2^	-	-	-	ns			
	Carbohydrate^2^	-	-	-	ns			
	Protein*Carbohydrate	0.020	0.006	3.1	0.0038			

The RSM analyses tested how the relative dietary content of protein, carbohydrates, and their interaction affect foraging response variables (harvested diet, consumed diet, hoarded diet, scattered diet), and colony performance response variables (percent surviving workers, number of eggs). Harvested diet was separated into hoarded (inside the nest), scattered (in the foraging area) and consumed (harvested–(hoarded + scattered)) components. Lipid effects on response variables reflect P:C:L diet recipes, such that high values of either protein or carbohydrates indicate low lipid values, low values of both protein and carbohydrates indicate high values of lipids, and low to medium values of both protein and carbohydrates indicate medium values of lipids. Thus, maximum response levels for lipids can be inferred by subtracting maximum responses for protein and carbohydrates from 100. The term ‘ns’ refers to non-significant treatment effects within RSM models that were removed from the final analyzed model. For the lack of fit statistic, non-significant p values (> 0.05) indicate the overall model adequately met the assumptions of the RSM. We did not interpret non-significant overall models for hoarded diet and scattered diet and thus did not include P:C:L landscapes based on these non-significant response variables.

Diet nutrient composition explained substantial variation in adult worker survival (55%, Table 1, Fig 4C) and colony growth rate (egg number, 23%, Table 1, Fig 4D). First, adult worker survival significantly increased to a maximum of > 70% of the initial workforce with rising carbohydrate content, although leveling out at P:C:L diets with ≥ 45% carbohydrates. Second, protein content did not significantly directly impact adult worker survival (Fig 4C, Table 1), but it modulated the effects of dietary carbohydrates, with the highest worker survival levels occurring at even levels of carbohydrate:lipid and low levels of protein (Fig 4C, Table 1). Third, protein lowered worker survival as it increased relative to carbohydrates (Fig 4C, Table 1, interaction effects). Fourth, while carbohydrate-rich diets increased adult worker survival, they had negative impacts on egg number, which decreased significantly in a linear fashion as carbohydrate increased (Fig 4D, Table 1). This negative carbohydrate effect on colony growth was strongest when protein simultaneously decreased (Fig 4D, Table 1, interaction effects).

These results support a tradeoff between colony performance traits since adult worker survival (highest value at 0:62:38 P:C:L; Table 1) and egg production (highest value at 57:43:0 P:C:L; Table 1) were maximized on different regions of the 3-D landscape surface (Fig 4C and 4D). Additionally, lipids did not appear to provide an alternative metabolic fuel resolving this growth-survival tradeoff since: 1) lipids had minor effects on adult survival (Fig 4C), 2) the highest colony growth occurred on diets with the lowest lipid levels (Fig 4D), and 3) lipids were not sufficient to rescue colony growth when comprising 80% of dietary macronutrients (i.e. when protein and carbohydrates were limiting) (Fig 4D, Table 1).

## Discussion

We combined NG paradigms to place a widespread food generalist on a generalist-specialist continuum of macronutrient regulation, while also linking its fundamental macronutrient niche (FMN) breadth with its colony founding performance. Using 2-D arrays, we show that *M*. *pharaonis* colonies exhibit a generalist equal-distance strategy of regulating total diet harvest and consumption levels even if this requires over-consuming both protein and carbohydrates relative to their respective intake targets. We next show that colonies navigate a 3-D P:C:L landscape by foraging broadly between carbohydrate-biased and protein-biased diets, while managing a tradeoff between worker survival (maximized by extreme C-biased diets) and colony egg production (maximized by balanced P:C diets). Lipids do not appear to suffice as an alternative energy source for colonies, since lipid-biased diets did not enable colonies to avert this survival-growth tradeoff under P and C limitation. An important next step will be to test whether and how the realized macronutrient niche of *M*. *pharaonis* approaches its FMN over time during invasions as colony demographic demands change [5], and across invasive populations confined to different nutritional environments [43].

Our results suggest that workers of *M*. *pharaonis* overharvest protein in ways that prioritize colony growth over individual survival when present in small isolated propagules, which may enable colonies to overcome founding bottlenecks en route to invasive success [31]. Comparative studies with non-invasive *Monomorium* species will be useful to test whether *M*. *pharaonis* uniquely prioritizes colony growth, and whether such strategies are more likely to evolve in invasive ants like *M*. *pharaonis* with sterile workers and massive colonies that only propagate as isolated lineages (e.g., [44]). Moreover, the broad FMN of *M*. *pharaonis* may also reflect its specialized existence limited to the human-built world, where plentiful food subsidies likely provide unusual and widely varying nutritional blends relative to foods available in natural habitats [45,46]. More generally, the invasive ant species studied to date further appear more likely to exhibit generalist macronutrient foraging strategies (i.e. the equal distance rule [26,30]) than non-invasive ant species exhibiting closest distance strategies ranging from prioritizing carbohydrate regulation (generalist scavenger *Rhytidoponera* sp. [25]) to protein regulation (fungus-farming detritivore, *Mycocepurus smithii* [42]). In this context, NG techniques will likely be useful for detecting strategies of nutrient regulation unique not only to invasive species, but also unique to specific invasive strategies.

More generally, colonies of *M*. *pharaonis* share a suite of behavioral traits (i.e. fast discovery and dominance of resources) and life history traits (i.e. many queens, reproduction by colony budding) with other successful invasive ants [16,47,48]. Here, we show an additional trait favoring invasiveness—the tendency to overharvest nutritionally variable resources relative to immediate colony needs, which ensures stored food availability in a fluctuating environment, while also lowering food available to potential competitors. A study of *Solenopsis invicta*, an invasive ant closely-related to *Monomorium*, found that it couples an overharvesting strategy with targeted hoarding of protein and consumption of carbohydrates to consume a narrow subset of harvested nutrients [30]. *Monomorium pharaonis* may have a broader FMN since it generally consumes most of the food it collects and in macronutrient blends matching those it harvests. An exception was in the 2-D choice experiment, where colonies exhibited slightly higher levels of carbohydrate-biased diet hoarding and scattering, although these post-processing amounts were a small fraction of harvested diet and were not sufficient to alter the intake target among diet pairing treatments. We note however, that we did not analyze the nutritional content of hoarded and scattered diet, so are unable to determine whether ants may have manipulated harvested diet in subtle ways to selectively save or dispose of specific nutrients within diets, as was seen in *Rhytidoponera* sp. as their colony demography changed [25].

By using the recently developed right-angled mixture approach [22] for the first time to study a social insect, we provide a new way to visualize an often cited (but seldom demonstrated) nutritional tradeoff between ant colony growth (protein-biased) and adult worker survival (carbohydrate biased) [28,29]. Specifically, we demonstrate that the carbohydrate-rich P:C:L blends maximizing adult survival performance do not overlap with more protein-rich regions maximizing egg production, while also showing that *M*. *pharaonis* colonies forage for broadly variable P:C blends spanning these performance maxima (Fig 4). This result echoes nutritional foraging tradeoffs faced by female *D*. *melanogaster* fruit flies that prioritize maximizing egg-laying rates (maximized by 1:2 P:C) over their own lifespans (maximized by 1:16 P:C) [49]. However, it is possible that sterile *M*. *pharaonis* workers have more latitude to navigate nutritional landscapes while avoiding longevity-reproduction tradeoffs, since they regurgitate harvested proteins to provision developing brood produced by the queen, rather than assimilating (as in the flies) them to sustain their own internally developing eggs.

NG studies have increasingly demonstrated the power of exploring the interactive effects of nutrient co-limitation on organism performance, rather than focusing on any single nutrient in isolation [7]. For instance, while little is known about how lipid requirements may interact with P:C requirements, the P:C:L landscapes used here show that dietary lipids did not limit worker mortality on protein-biased diets or facilitate colony growth on carbohydrate-biased diets. Thus, lipids do not appear interchangeable with carbohydrates as energy sources that maximize colony performance [23]. This is somewhat surprising since *M*. *pharaonis* is known to forage oils (e.g., peanut oil) in single-food feeding experiments [44,50], since many sterols and unsaturated fatty acids cannot be synthesized by insects [51], and since lipids can generally provide important metabolic fuel during periods of food scarcity [52], during insect growth and development [53], and during ant mating flights [54]. Additionally, ant colony nutritional requirements may also interact with seasonal variation in nutritional foraging decisions [55–57] that may in turn reflect the specific nutritional demands of annual life history cycles. For instance, a colony’s allocation to increased colony growth and reproduction can deplete the fat stores in adult workers [58,59], and this may in turn influence the decisions of foragers to harvest and hoard lipids. The integration of such longer term colony dynamics will be an important next step. Finally, beyond adding new macronutrients to longitudinal NG studies of single colonies in the field, additional mechanistic insights will be gained by also parsing carbohydrates among diverse energetic carbon sources [60], proteins into specific amino acids [61], and selecting lipids from the broad chemical and functional diversity to test specific hypotheses.

## Supporting information

S1 Fig **Colonies regulate A) total diet harvest levels, while allowing their harvest of (A) carbohydrate and (B) protein to fluctuate.** Cumulative dry diet mass amounts (± SE) was harvested by colonies over 12 days during the 2-D no-choice P:C diet experiment. Colonies consistently harvested similar amounts of diet across P:C diet treatments, and thus harvested more carbohydrates on high-carbohydrate diets, and more protein on high-protein diets. Letters indicate significant differences (p < 0.05) among diet treatments, as determined by post-hoc Tukey tests.
(PDF)

S2 Fig **Colonies (A) consumed more on protein-biased diets, while also overconsuming both (B) carbohydrates and (C) protein when either was provided in overabundance in the 2-D no-choice feeding experiment.** Mean values of cumulative diet consumption (harvested–(hoarded + scattered)) over 12 days (± SE) are presented, with letters indicating significant differences (p < 0.05) among diet treatments, as determined by post-hoc Tukey tests.
(PDF)

S3 Fig **Worker survival curves over 12 days for the (A) no-choice and (B) choice 2-D diet P:C feeding experiments.** Within both experiments, worker survivorship was lower when workers were confined to protein-biased diets. However, worker survivorship was significantly higher when workers could select their own intake target in the choice experiment relative to the no-choice experiment.
(PDF)

S4 Fig Stacked bar graph comparing how fractions of harvested diet (masses (mg) of consumed, hoarded, scattered) differed across colonies confined to different P:C:L diets over 14 days. White letters in black bars indicate significant *consumed diet* Tukey-test groupings based on significant GLM analysis, and black letters above bars indicate significant *harvested diet* Tukey-test groupings based on GLM analysis.
(PDF)

S1 Table Diet recipes for 2-D feeding experiment. We devised a modified version of the nutritionally defined protein:carbohydrate (P:C) diet of [31] with a 100 g/L protein plus carbohydrate dilution. For preparation details, see Methods and S1 Appendix. Values in parentheses indicate the amount of protein provided by the ingredient as specified on ingredient labels. Small amounts of carbohydrates provided by egg powder (2.00%) and calcium caseinate (1.89%) were also incorporated into diet recipes. All amounts are provided in grams (g), with 30 g protein + carbohydrates prepared in 300 ml of demineralized H_2_O.
(PDF)

S2 Table Diet recipes for 3-D feeding experiment. We prepared diets using modified versions of a protein:carbohydrate:lipid (P:C:L) diet used by [23]. For preparation details, see Methods and S1 Appendix. All diets standardized so they provided *ca*. 675 joules from the combined amounts of protein, carbohydrates, and lipids they provided, while manipulating the relative joules provided by each macronutrient, assuming that lipids contain twice the amount of energy as carbohydrates and proteins. Recipes were constructed from ingredient labels, assuming whey protein had 6.7% fat and 80% protein, calcium caseinate had 1.2% fat and 93.5% protein and egg white protein had 96% protein per unit dry mass. All amounts are provided in grams (g), with 30 g protein + carbohydrates prepared in 300 ml of demineralized H_2_O. Sucrose was used as the carbohydrate source, and dried egg white powder, whey protein and calcium caseinate were used as the protein source in approximately 1:1:1 ratio and lipids were provided by a ca. 4:1:1:1:1 ratio of lard:fish-oil:sunflower-oil:rapeseed-oil:peanut-oil. Lard was melted, mixed with the other oils, and then combined with 2 ml of chloroform. This mixture was combined with the dry ingredients and the chloroform was allowed to evaporate under a fume hood at room temperature for 96 hr (*as per* [23]).
(PDF)

S3 Table Statistical output from general linear model (GLM) analyses testing how the relative dietary content of protein, carbohydrates, and their interaction affect four foraging response variables. Significant analyses followed up with post-hoc Tukey tests, displayed in S4 Fig.
(PDF)

S1 Appendix Detailed information on: 1) the experimental setup, 2) diet preparation, 3) the 2-D nutritional geometry feeding experiment, and 4) the 3-D nutritional geometry experiment. (PDF)
