# Supplementary material for: Using nutritional geometry to define the fundamental macronutrient niche of the widespread invasive ant Monomorium pharaonis
Source: PLoS One. 2019 Jun 20;14(6):e0218764. doi: 10.1371/journal.pone.0218764 (PMC6586327; doi:10.1371/journal.pone.0218764)
Supplement: S1 Fig — Colonies regulate A) total diet harvest levels, while allowing their harvest of (A) carbohydrate and (B) protein to fluctuate. Cumulative dry diet mass amounts (± SE) was harvested by colonies over 12 days during the 2-D no-choice P:C diet experiment. Colonies consistently harvested similar amounts of diet across P:C diet treatments, and thus harvested more carbohydrates on high-carbohydrate diets, and more protein on high-protein diets. Letters indicate significant differences (p < 0.05) among diet treatments, as determined by post-hoc Tukey tests. (PDF) [file pone.0218764.s001.pdf]

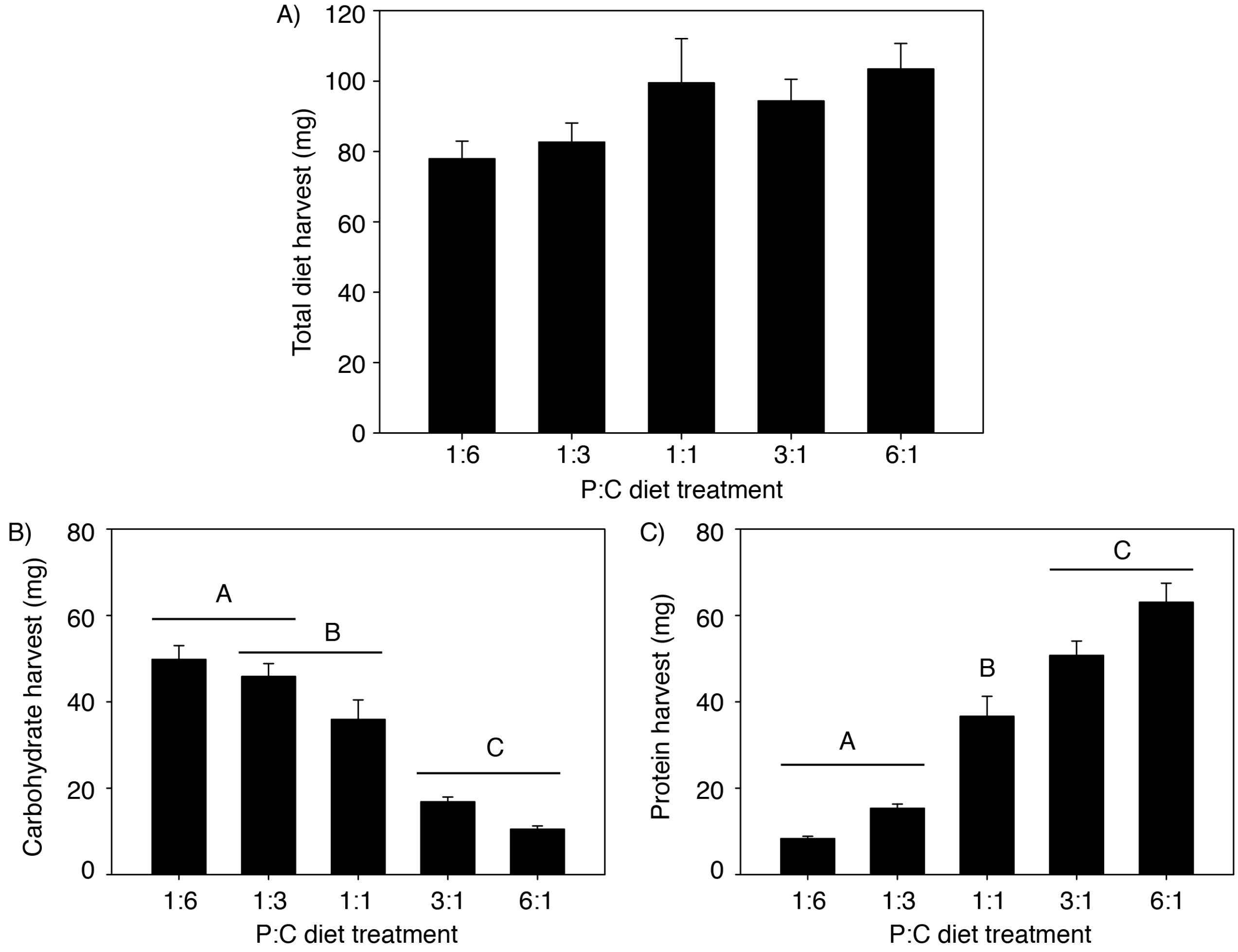

Figure S1 Colonies regulate A) total diet harvest levels, while allowing their harvest of A) carbohydrate and B) protein to fluctuate. Cumulative dry diet mass amounts (± SE) was harvested by colonies over 12 days during the 2-D no-choice P:C diet experiment. Colonies consistently harvested similar amounts of diet across P:C diet treatments, and thus harvested more carbohydrates on high-carbohydrate diets, and more protein on high-protein diets. Letters indicate significant differences ($p < 0.05$) among diet treatments, as determined by post-hoc Tukey tests.
