# Supplementary material for: Using nutritional geometry to define the fundamental macronutrient niche of the widespread invasive ant Monomorium pharaonis
Source: PLoS One. 2019 Jun 20;14(6):e0218764. doi: 10.1371/journal.pone.0218764 (PMC6586327; doi:10.1371/journal.pone.0218764)
Supplement: S2 Fig — Colonies (A) consumed more on protein-biased diets, while also overconsuming both (B) carbohydrates and (C) protein when either was provided in overabundance in the 2-D no-choice feeding experiment. Mean values of cumulative diet consumption (harvested–(hoarded + scattered)) over 12 days (± SE) are presented, with letters indicating significant differences (p < 0.05) among diet treatments, as determined by post-hoc Tukey tests. (PDF) [file pone.0218764.s002.pdf]

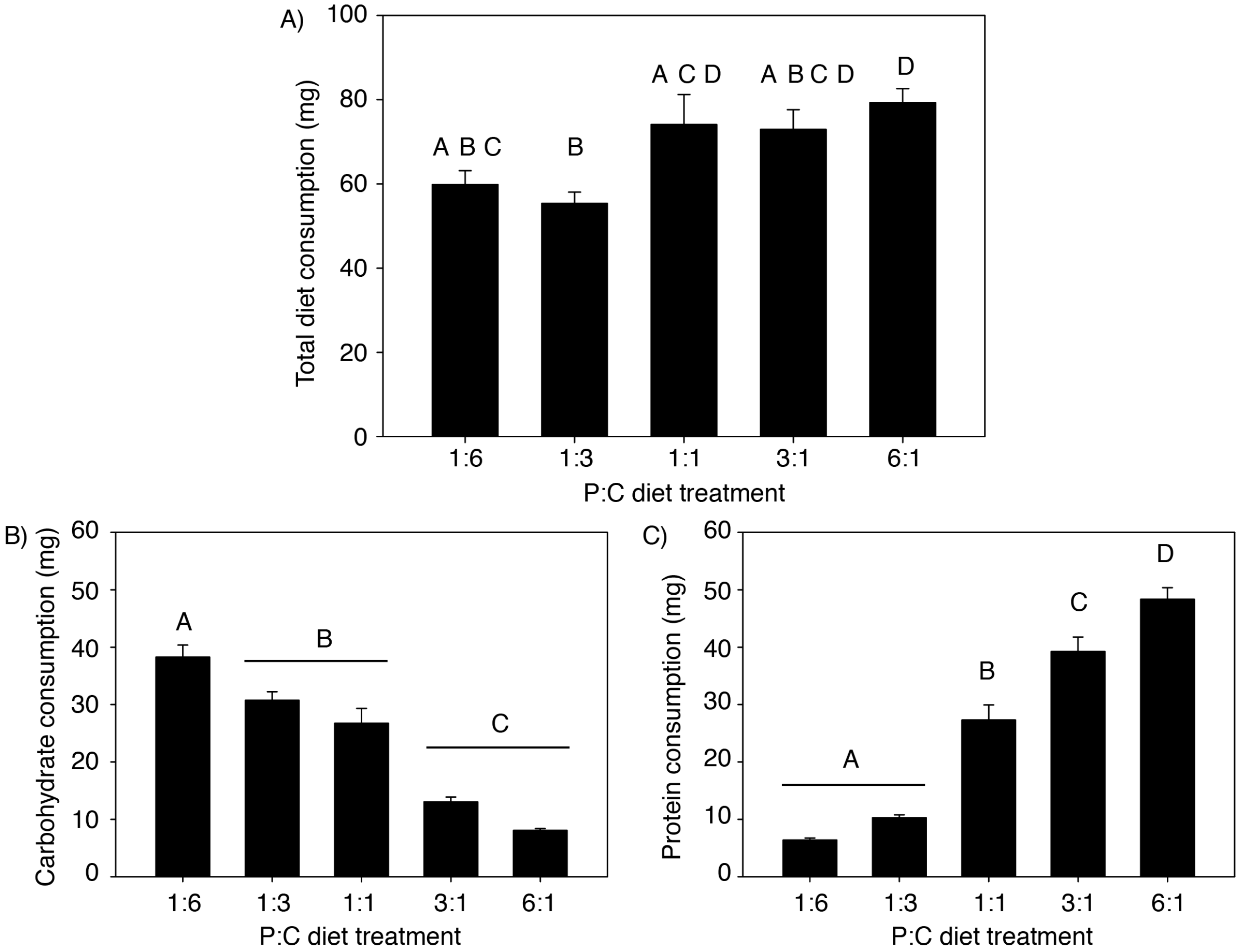

Figure S2 Colonies A) consumed more on protein-biased diets, while also overconsuming both B) carbohydrates and C) protein when either was provided in overabundance in the 2-D no-choice feeding experiment. Mean values of cumulative diet consumption (harvested – (hoarded + scattered)) over 12 days (± SE) are presented, with letters indicating significant differences ($p < 0.05$) among diet treatments, as determined by post-hoc Tukey tests.
