# Supplementary material for: Using nutritional geometry to define the fundamental macronutrient niche of the widespread invasive ant Monomorium pharaonis
Source: PLoS One. 2019 Jun 20;14(6):e0218764. doi: 10.1371/journal.pone.0218764 (PMC6586327; doi:10.1371/journal.pone.0218764)
Supplement: S3 Fig — Worker survival curves over 12 days for the (A) no-choice and (B) choice 2-D diet P:C feeding experiments. Within both experiments, worker survivorship was lower when workers were confined to protein-biased diets. However, worker survivorship was significantly higher when workers could select their own intake target in the choice experiment relative to the no-choice experiment. (PDF) [file pone.0218764.s003.pdf]

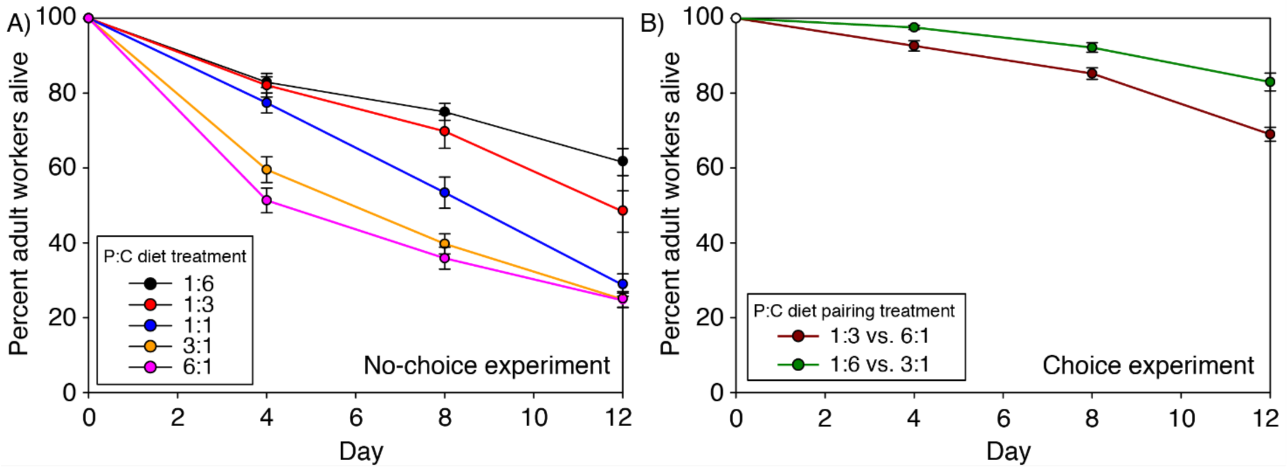

Figure S3 Worker survival curves over 12 days for the a) no-choice and b) choice 2-D diet P:C feeding experiments. Within both experiments, worker survivorship was lower when workers were confined to protein-biased diets. However, worker survivorship was significantly higher when workers could select their own intake target in the choice experiment relative to the no-choice experiment.
