# Supplementary material for: Using nutritional geometry to define the fundamental macronutrient niche of the widespread invasive ant Monomorium pharaonis
Source: PLoS One. 2019 Jun 20;14(6):e0218764. doi: 10.1371/journal.pone.0218764 (PMC6586327; doi:10.1371/journal.pone.0218764)
Supplement: S4 Fig — White letters in black bars indicate significant consumed diet Tukey-test groupings based on significant GLM analysis, and black letters above bars indicate significant harvested diet Tukey-test groupings based on GLM analysis. (PDF) [file pone.0218764.s004.pdf]

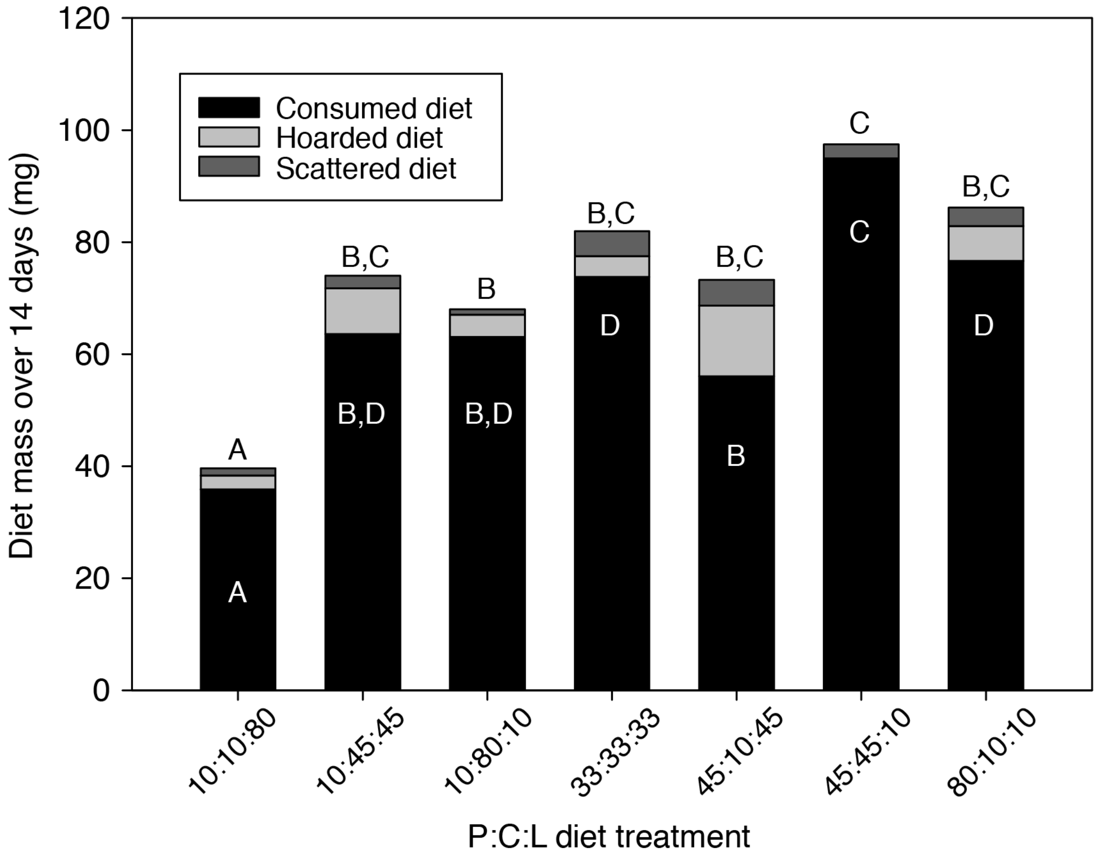

Figure S4 Stacked bar graph comparing how fractions of harvested diet (masses (mg) of consumed, hoarded, scattered) differed across colonies confined to different P:C:L diets over 14 days. White letters in black bars indicate significant *consumed diet* Tukey-test groupings based on significant GLM analysis, and black letters above bars indicate significant *harvested diet* Tukey-test groupings based on GLM analysis.
