# Supplementary material for: Using nutritional geometry to define the fundamental macronutrient niche of the widespread invasive ant Monomorium pharaonis
Source: PLoS One. 2019 Jun 20;14(6):e0218764. doi: 10.1371/journal.pone.0218764 (PMC6586327; doi:10.1371/journal.pone.0218764)
Supplement: S1 Table — We devised a modified version of the nutritionally defined protein:carbohydrate (P:C) diet of [31] with a 100 g/L protein plus carbohydrate dilution. For preparation details, see Methods and S1 Appendix. Values in parentheses indicate the amount of protein provided by the ingredient as specified on ingredient labels. Small amounts of carbohydrates provided by egg powder (2.00%) and calcium caseinate (1.89%) were also incorporated into diet recipes. All amounts are provided in grams (g), with 30 g protein + carbohydrates prepared in 300 ml of demineralized H2O. (PDF) [file pone.0218764.s005.pdf]

Table S1 We devised a modified version of the nutritionally defined protein:carbohydrate (P:C) diet of [31] with a 100 g/L protein plus carbohydrate dilution. For preparation details, see Methods and Appendix S1. Values in parentheses indicate the amount of protein provided by the ingredient as specified on ingredient labels. Small amounts of carbohydrates provided by egg powder (2.00%) and calcium caseinate (1.89%) were also incorporated into diet recipes. All amounts are provided in grams (g), with 30 g protein + carbohydrates prepared in 300 ml of demineralized H_2O.

	P:C ratio				
	6:1	3:1	1:1	1:3	1:6
Ingredients:					
H_2O (ml)	300.00	300.00	300.00	300.00	300
Dried egg powder (g)	9.00 (4.23)	9.00 (4.23)	9.00 (4.23)	9.00 (4.23)	9.00 (4.23)
Whey protein (g)	11.31 (10.15)	9.72 (8.72)	5.82 (5.22)	1.73 (1.55)	0.033 (0.03)
Calcium Caseinate (g)	12.41 (11.36)	10.56 (9.66)	6.39 (5.85)	1.89 (1.73)	0.036 (0.03)
Sucrose (g)	3.89	7.14	14.70	22.26	25.53
Vanderzant vitamin mixture	0.81	0.81	0.81	0.81	0.81
Agar (g)	4.80	4.80	4.80	4.80	4.80
