# Supplementary material for: Using nutritional geometry to define the fundamental macronutrient niche of the widespread invasive ant Monomorium pharaonis
Source: PLoS One. 2019 Jun 20;14(6):e0218764. doi: 10.1371/journal.pone.0218764 (PMC6586327; doi:10.1371/journal.pone.0218764)
Supplement: S3 Table — Significant analyses followed up with post-hoc Tukey tests, displayed in S4 Fig. (PDF) [file pone.0218764.s007.pdf]

Table S3 Statistical output from general linear model (GLM) analyses testing how the relative dietary content of protein, carbohydrates, and their interaction affect four foraging response variables. Significant analyses followed up with post-hoc Tukey tests, displayed in Figure S4.

Response variable	Variable	Df	Sum Squares	F	P
Harvested diet	Diet	6	9890	10.84	< 0.0001
	Residuals	28	4258		
Consumed diet	Diet	6	10106	23.49	< 0.0001
	Residuals	28	2007		
Hoarded diet	Diet	6	504	1.08	0.396
	Residuals	28	2172		
Scattered diet	Diet	6	5.641	2.21	< 0.072
	Residuals	28	11.927		
