# Supplementary material for: Using nutritional geometry to define the fundamental macronutrient niche of the widespread invasive ant Monomorium pharaonis
Source: PLoS One. 2019 Jun 20;14(6):e0218764. doi: 10.1371/journal.pone.0218764 (PMC6586327; doi:10.1371/journal.pone.0218764)
Supplement: S2 Table — We prepared diets using modified versions of a protein:carbohydrate:lipid (P:C:L) diet used by [23]. For preparation details, see Methods and S1 Appendix. All diets standardized so they provided ca. 675 joules from the combined amounts of protein, carbohydrates, and lipids they provided, while manipulating the relative joules provided by each macronutrient, assuming that lipids contain twice the amount of energy as carbohydrates and proteins. Recipes were constructed from ingredient labels, assuming whey protein had 6.7% fat and 80% protein, calcium caseinate had 1.2% fat and 93.5% protein and egg white protein had 96% protein per unit dry mass. All amounts are provided in grams (g), with 30 g protein + carbohydrates prepared in 300 ml of demineralized H2O. Sucrose was used as the carbohydrate source, and dried egg white powder, whey protein and calcium caseinate were used as the protein source in approximately 1:1:1 ratio and lipids were provided by a ca. 4:1:1:1:1 ratio of lard:fish-oil:sunflower-oil:rapeseed-oil:peanut-oil. Lard was melted, mixed with the other oils, and then combined with 2 ml of chloroform. This mixture was combined with the dry ingredients and the chloroform was allowed to evaporate under a fume hood at room temperature for 96 hr (as per [23]). (PDF) [file pone.0218764.s006.pdf]

Table S2 We prepared diets using modified versions of a protein:carbohydrate:lipid (P:C:L) diet used by [23]. For preparation details, see Methods and Appendix S1. All diets standardized so they provided *ca.* 675 joules from the combined amounts of protein, carbohydrates, and lipids they provided, while manipulating the relative joules provided by each macronutrient, assuming that lipids contain twice the amount of energy as carbohydrates and proteins. Recipes were constructed from ingredient labels, assuming whey protein had 6.7% fat and 80% protein, calcium caseinate had 1.2% fat and 93.5% protein and egg white protein had 96% protein per unit dry mass. All amounts are provided in grams (g), with 30 g protein + carbohydrates prepared in 300 ml of demineralized H_2O. Sucrose was used as the carbohydrate source, and dried egg white powder, whey protein and calcium caseinate were used as the protein source in approximately 1:1:1 ratio and lipids were provided by a ca. 4:1:1:1:1 ratio of lard:fish-oil:sunflower-oil:rapeseed-oil:peanut-oil. Lard was melted, mixed with the other oils, and then combined with 2 ml of chloroform. This mixture was combined with the dry ingredients and the chloroform was allowed to evaporate under a fume hood at room temperature for 96 hr (*as per* [23]).

	P:C:L diets						
	33:33:33	10:80:10	80:10:10	10:10:80	45:45:10	45:10:45	10:45:45
Ingredients:							
H_2O (ml)	300.00	300.00	300.00	300.00	300.00	300.00	300
Dried egg white powder (g)	5.13	1.67	12.16	1.67	7.46	6.77	1.67
Whey protein (g)	5.10	1.66	12.07	1.66	7.25	6.73	1.66
Calcium Caseinate (g)	4.86	1.47	11.16	1.47	6.63	6.63	1.47
Sucrose (g)	12.96	31.54	3.10	4.05	18.44	3.49	18.46
Animal fat (g)	2.93	0.87	0.45	8.06	0.66	3.93	4.53
fish oil (g)	0.70	0.21	0.10	1.77	0.16	0.95	0.99
peanut oil (g)	0.70	0.21	0.10	1.77	0.16	0.95	0.99
rapeseed oil (g)	0.70	0.21	0.10	1.77	0.16	0.95	0.99
sunflower oil (g)	0.70	0.21	0.10	1.77	0.16	0.95	0.99
Vanderzant vitamin mixture	0.81	0.81	0.81	0.81	0.81	0.81	0.81
Agar (g)	4.80	4.80	4.80	4.80	4.80	4.80	4.80
