# Supplementary material for: Using nutritional geometry to define the fundamental macronutrient niche of the widespread invasive ant Monomorium pharaonis
Source: PLoS One. 2019 Jun 20;14(6):e0218764. doi: 10.1371/journal.pone.0218764 (PMC6586327; doi:10.1371/journal.pone.0218764)
Supplement: S1 Appendix — (PDF) [file pone.0218764.s008.pdf]

Supplementary Appendix S1

Experimental setup

We performed laboratory nutritional geometry feeding experiments on small *Monomorium pharaonis* propagules generated by taking ants from a mixed lab population originally collected from source populations in Florida (n = 2 populations), Texas (n = 2 populations), Malaysia (n = 1), London (n = 1), Warsaw (n = 1), and Ghana (n = 1) that span the global distribution of this species [1,2]. Sub-colonies of ants blended from these poopulations have subsequently been maintained long-term on *ad lib* amounts of crickets and sugar-agar diet (*modified from* [3]). The present study exploring the effects of nutrient co-limitation builds upon earlier single resource manipulation laboratory feeding experiments showing that *M. pharaonis* workers recruited more strongly to carbohydrate-rich foods (e.g. 1 M sucrose) and lipid-rich foods (e.g. peanut oil) and stored protein-rich foods (moist egg-yolk powder) which was subsequently detected more heavily in older larvae [4], and that *M. pharaonis* foraging responses may differ from other invasive congeneric *Monomorium* species [5].

Ants were selected from 6 mixed colony bins in the 2-D experiment, and from 8 mixed colony bins in the 3-D experiment. For the 2-D experiment, all experiments were performed in climate-controlled rooms at 27°C and 50% R.H. In the acclimation period prior to feeding experiments (4 days for 2-D, 9-11 days for 3-D), workers, queens and brood (amounts specified below), were removed from source colonies with a small paintbrush and placed in 14 cm diameter petri dish arenas with fluon coated walls placed on mesh platforms over a glycerin filled tray. In the 2-D experiment, colonies were further limited to an 11 cm diameter area within petri dishes (by a fluon-coated plexiglass cylinder), and the floor was covered with filter paper.

Colonies relocated to defined nesting areas within hours of placement in arenas, which consisted of a black cardboard rectangle (3.5 x 2.5 cm) elevated slightly by a folded corner in the 2-D experiment, or a 4 cm^2 square with a small entrance, and a transparent red cellophane square covered by glass in the 3-D experiment. Lids containing pre-weighed experimental foods were placed in the foraging area near the nest. In the 2-D experiment, colonies were provided a fresh block (ca. 1 cm^3) of demineralized water mixed with agar (16g/L) each day. Agar-water cubes (16 g/L) were used since we expected workers to scatter bits of cotton from traditional cotton-plugged water tubes in foraging areas, that may have hampered recovery of scattered diet (*see below*). However, we switched back to water tubes for the 3-D experiment when it became clear that scattered diet could be easily distinguished from small discarded cotton bits. To provide water, colonies were provided a fresh block (ca. 1 cm^3) of demineralized water mixed with agar (16g/L) each day (2-D experiment), or water tubes (3-D experiment).

Diet preparation

Diets were prepared using modified versions of a published protein:carbohydrate (P:C) diet [3] and a protein:carbohydrate:lipid (P:C:L) diet [6]. The P:C diet contained protein from whey powder (Myopure), whole egg powder (Great American Spice Company), and calcium caseinate (Arla), carbohydrates from sucrose (Sigma Aldrich), and micronutrients from Vanderzant vitamin mixture (Sigma Aldrich). Sucrose was used as the carbohydrate source, and dried egg white powder, whey protein and calcium caseinate were used as the protein source in approximately 1:1:1 ratio. The P:C:L diet used the same ingredients, but replaced whole-egg powder with egg-white only powder (Myoprotein), and provided lipids with a 4:1:1:1:1 ratio of lard:fish-oil:sunflower-oil:rapeseed-oil:peanut-oil. This mixture was chosen based on a series of

pilot experiments showing that workers recruited most to lard, followed by similar recruitment levels to the 4 selected oils relative to 3 other less preferred oils (safflower oil, olive oil, coconut oil). Lard was melted, mixed with the other oils, and then combined with 2 ml of chloroform. This mixture was combined with the dry ingredients and the chloroform was allowed to evaporate under a fume hood at room temperature for 96 hr (*as per* [6]). Diet recipes are provided in Table S1 and Table S2.

To prepare diets, agar was gently heated while stirring in water (16 g/L) until boiling and was then cooled slightly before being mixed in a blender with pre-weighed macronutrient ingredients. Mixed diets were then poured into petri dishes, sealed with parafilm, and stored at 4° C until provided to ants. For all experiments, we measured diet harvest by placing pre-weighed (initial wet mass) diet cubes (*ca.* 1 cm^3) on small dishes inside colony foraging areas, and then collecting them after 24 hours. These diets were then oven-dried at 60°C for 24 hours and weighed to the nearest 1 μg (final dry mass) on an AG285 Mettler Toledo microbalance. Each day, we also recorded wet and dry mass of 4 control cubes of each P:C diet, which enabled us to calculate dry:wet conversion factors used to estimate initial dry mass of each experimental diet cube [7]. We summed these diet values to calculate cumulative harvest for the experiment.

We also added a few drops of food coloring (Dr. Oetker ™) to each diet just prior to blending all ingredients, which enabled us to separate it from debris when collecting hoarded (piled within the defined nest area) and scattered (discarded in the foraging area) diet at the end of the experiment. In the 2-D choice experiment, we further colored the two diets differently so that we could distinguish them to reconstruct macronutrient compositions in subsequent analyses. While diet color did not impact colony foraging behavior in pilot experiments, we nonetheless varied the color-nutrient combinations (i.e. the 1:6 diet was blue and 3:1 diet was red

in one pairing and reversed in another pairing). Hoarded and scattered diets were oven dried and then weighed to the nearest 1 μg. Consumed diet was harvested diet minus the summed mass of hoarded and scattered diet.

2-D nutritional geometry experiment

Each colony was established with 200 workers and a scoop (0.5 x 0.5 x 0.15 cm) of brood, so that each colony had brood on day 1 of the experiment, and similar amounts of older brood which are known to be important for nutrient processing in *M. pharaonis* colonies [8,9]. Dead workers were collected from each colony during the four day acclimation period and were replaced on day 1 of the experiment to standardize initial colony size. Queens were not included in this experiment because we were interested in colony foraging decisions rather than colony growth performance. While *M. pharaonis* workers, lacking an egg laying queen, may have attempted to convert eggs or first instar larvae present in the initial scoop of brood into sexuals (Pontieri, *pers. comm.*), which may have altered their foraging behavior, we did not detect any sexual larvae or pupae in colonies during the 12-day experiment. We assigned 24 colonies to the choice experiment (n = 12 colonies per choice pairing treatment, but removed one colony from each choice treatment due to missing intake data on one day), leaving 11 colonies per choice pairing treatment. We also assigned 40 colonies to the no-choice experiment (n = 8 colonies per diet treatment). Over 12 days in both choice and no-choice experiments, we replaced old diet with fresh diet daily. We also counted and collected dead workers every fourth day during the experiment and collected the remaining living workers on day 12.

3-D nutritional geometry experiment

The P:C:L diet treatments did not significantly affect the number of larvae (explained 2% of variation in this response variable), although they explained a significant amount (23%) of variation in egg number (Table 1). This difference was likely due to larva number representing brood present during the acclimation phase (when all colonies received the same standard diet), but egg number representing queen health and activity during the colonies' exposure to P:C:L diet treatments. Thus, larva number was not considered further in the study. Additionally, P:C:L landscapes were not produced for scattered or hoarded diet results, since the overall RSM models were also not significant for these variables (Table 1).

References

1. Schmidt AM, d'Ettorre P, Pedersen J. Low levels of nestmate discrimination despite high genetic differentiation in the invasive pharaoh ant. Front Zool. 2010; 7:20.
2. Pontieri L, Schmidt AM, Singh R, Pedersen JS, Linksvayer TA. Artificial selection on ant female caste ratio uncovers a link between female-biased sex ratios and infection by Wolbachia endosymbionts. J Evol Biol. 2017; 30:225-234.
3. Dussutour A, Simpson SJ. Description of a simple synthetic diet for studying nutritional responses in ants. Ins Soc. 2008; 55:329-333.
4. Haack KD, Vinson SB, Olson JK. Food distribution and storage in colonies of *Monomorium pharaonis* (L.) (Hymenoptera: Formicidae). J Entomol Sci. 1995; 30:70-81.
5. Eow AGH, Lee CY. Comparative nutritional preferences of tropical pest ants, *Monomorium pharaonis*, *Monomorium floricola* and *Monomorium destructor* (Hymenoptera: Formicidae). Sociobiol. 2007; 49:165-186.

6. Hawley J, Simpson SJ, Wilder SM. Flesh flies regulate the consumption of 3 macronutrients to maximize lifespan and egg production. Behav Ecol. 2015; 27:245-251.
7. Kay AD, Shik JZ, Van Alst A, Kaspari M. Diet composition does not affect ant colony tempo. Funct Ecol. 2012; 26:317-323.
8. Børgesen LW, Jensen PV. Influence of larvae and workers on egg production of queens of the pharaoh's ant, *Monomorium pharaonis* (L.). Ins Soc. 1995; 42:103-112.
9. Warner MR, Kovaka K, Linksvayer TA. Late-instar ant worker larvae play a prominent role in colony-level caste regulation. Ins Soc. 2016; 63:575-583.
